# Awareness of nutrition and health knowledge and its influencing factors among Wuhan residents

**DOI:** 10.3389/fpubh.2022.987755

**Published:** 2022-10-05

**Authors:** Yating Wu, Shanshan Wang, Mengdie Shi, Xinzheng Wang, Hongjuan Liu, Shu Guo, Le Tan, Xuefeng Yang, Xiaomin Wu, Liping Hao

**Affiliations:** ^1^Institute of Environmental Health and Food Safety, Wuhan Centre for Disease Control and Prevention, Wuhan, China; ^2^Department of Nutrition and Food Hygiene, Hubei Key Laboratory of Food Nutrition and Safety, MOE Key Laboratory of Environment and Health, School of Public Health, Tongji Medical College, Huazhong University of Science and Technology, Wuhan, China

**Keywords:** nutrition awareness, influencing factors, sociodemographic characteristics, health literacy, survey

## Abstract

**Background:**

Nutrition and health knowledge play a crucial role in promoting healthy dietary behaviors, and have been found to be related to sociodemographic characteristics. However, the existing evidence is limited and inconsistent. We aimed to evaluate the awareness level of nutrition and health knowledge and its influencing factors among Wuhan residents, and to provide scientific basis for carrying out targeted nutrition education programmes.

**Methods:**

By stratified random sampling, residents aged 18–64 in Wuhan were selected for self-administered questionnaire survey. We adopted the structured questionnaire to investigate respondents' sociodemographic characteristics, nutrition and health knowledge, and the way to acquire knowledge. Among them, nutrition and health knowledge includes the following four parts: dietary guidelines recommendations, food and nutrients, nutrition and disease prevention, and nutrition skills. Chi-square tests were used to analyze the associations between total awareness rate and sociodemographic characteristics. Multiple linear regression models were used to analyze the influencing factors of nutrition and health awareness.

**Results:**

A total of 33,436 valid questionnaires were obtained, with a response rate of 97.8%. The total awareness rate was 20.4%, with the highest in nutrition and disease prevention (72.7%) and the lowest in nutrition skills (46.3%). Responders aged 35–44 (23.3%), females (22.8%), educational workers (24.8%), obtaining a master's degree or above (34.1%), living in downtown area (23.1%), and without a history of chronic disease (24.6%) were more likely to have higher awareness rates (all *p* < 0.001). The multiple linear regression models showed that age, gender, education level, occupation, residential address, and the history of chronic disease were the potential factors affecting individual nutrition awareness.

**Conclusion:**

The total awareness rate of nutrition and health knowledge among Wuhan residents was not optimistic. Besides, our findings suggested that sociodemographic characteristics are closely related to nutrition awareness, which may provide important clues for carried out nutrition education campaigns.

## Introduction

With the rapid economic development and the improvement of living standards, significant changes have taken place in the lifestyle and dietary patterns of Chinese people. Meanwhile, the prevalence of nutrition-related chronic diseases such as obesity, hypertension and diabetes has increased year by year, causing huge burden on individuals and society (1, 2). Current evidence strongly suggests that dietary and nutritional status plays a critical role in the development of chronic diseases (3). However, date from the latest China Health and Nutrition Survey (1, 2, 4) revealed a series of nutrition-related problems in Chinese population: (a) the level of nutrition and health knowledge among Chinese residents varies greatly, and most people maintain unreasonable dietary habits; (b) unreasonable diet structure and unbalanced nutrition intake are common; and (c) more seriously, the dietary pattern of Chinese people is gradually shifting from a plant-based diet to a high-energy, high-fat and high-protein dietary pattern. The reasons for the above problems may be partly attributed to lower nutrition literacy, incorrect nutrition concepts and lack of relevant nutrition skills (5, 6). According to the knowledge, attitude and practice (KAP) model, acquiring knowledge is a key first step to promoting belief and changing undesirable behaviors (7). Therefore, given the potential health hazards caused by the above problems, there is an urgent need to find out the shortcomings of people's nutrition and health knowledge, and further improve people's nutrition and health literacy.

It is well accepted that a higher level of nutrition knowledge contributes to promoting healthier dietary behaviors, while a lower level of nutrition knowledge is closely related to poor eating habits, unbalanced dietary patterns (5, 6), and a higher risk of nutrition-related chronic diseases (8), since those who have a lower level of nutrition knowledge but perceive themselves as healthy consumers have no incentive to change their poor behavior (2, 9). Specifically, a randomized controlled trial conducted in sub-Saharan African compared the effects of supplementary nutrition education and dietary counseling on nutrition knowledge and dietary behavior among Malawian pregnant women, and found that the nutrition awareness and dietary behavior of the intervention group receiving nutrition and dietary guidance were significantly improved compared with the control group (10). Similarly, Bottcher et al. (11) pointed out that college students who received formal nutrition education were more likely to adhere to the Mediterranean diet. Moreover, a growing body of literature shows that there is a significant positive correlation between nutrition knowledge and favorable dietary-related behaviors, such as healthy weight loss (12), purchasing intention of nutritious claims (13), consuming more green vegetables (14), grains and dairy products (15). However, other studies did not find any associations between nutrition knowledge and healthier food choices (16) and healthy effects (17), suggesting that nutrition knowledge may be a necessary but insufficient factor to change people's behavior (18).

Individual sociodemographic characteristics, including age, education level, economic status, and residential area, are key factors affecting their nutrition and health knowledge (19, 20). However, due to the discrepancies in the study population, survey design and analysis methods, some available results on the relationship between sociodemographic characteristics and nutrition and health knowledge remain controversial (7, 21, 22). For instance, a cross-sectional study conducted in Nanjing, China found female students have a higher level of nutrition knowledge than male students (7). Whereas, Xu et al. (21) did not observe any relationship between nutrition knowledge and gender. With respect to the relationship between nutrition knowledge and age, the National Health and Nutrition Examination Survey 2005–2006 reported a negative linear correlation (22), while other studies did not observe any association (23, 24). Despite the contribution of nutrition and health knowledge to the change of dietary behavior is intricate and affected by various sociodemographic and environmental factors, a better understanding of the relationship between nutrition and health knowledge and its possible influencing factors is urgently needed.

To date, however, there are few large-scale studies on nutrition and health knowledge of the Chinese population and its possible socio-demographic factors. Thus, we aimed to acquire the level of nutrition and health knowledge of Wuhan residents and to explore its influencing factors. Our findings will help to provide scientific basis for carrying out targeted nutrition education programmes.

## Materials and methods

### Study design and participants

The China Nutrition and Health Knowledge Survey is an ongoing cross-sectional study launched in April 2021 in 31 provinces, cities and autonomous regions in China. The survey aims to explore the awareness level and its influencing factors of nutrition and health knowledge among Chinese adult residents and providing a scientific basis for the policy decision on nutrition intervention. Wuhan, a representative city in central China, conducted part of the survey from April 2021 to October 2021, and the permanent residents aged 18–64 from Wuhan were included in the analysis. Based on the results from the sixth national population census in China (25), we adopted the method of stratified cluster sampling, and selected participants according to the proportion of half male and half female, and the proportion of 20, 20, 20, 30, and 10% for 18–24, 25–34, 45–54, 35–44 and 55–64 years, respectively. The respondents were selected from 15 monitoring sites (3–10 communities were selected from each monitoring site). According to the population size of each monitoring site, 1–15 communities were selected for each monitoring site, and it is planned to survey 330 residents in each community, who were representative in terms of gender, age and regional distribution. After excluding people who refused to participate in the project, 33,436 of the 34,190 respondents were included in the analysis, with a response rate of 97.8%. The actual distribution of each monitoring site is shown in Supplementary Table S1. All respondents provided their written informed consent before conducting the survey. This study was approved by the Ethics Review Committee of the Wuhan Center for Disease Control and Prevention (approval number: WHCDCIRB-K-2021033).

### Questionnaire

The questionnaire was designed by the National Institute for Nutrition and Health, Chinese Center for Disease Control and Prevention, and formed after expert review, pre-survey, reliability and validity test. We adopted the structured questionnaire to investigate respondents' sociodemographic characteristics (age, gender, educational level, occupation, residential address, and history of chronic disease), nutrition and health knowledge, and the way to acquire knowledge.

The nutrition and health knowledge consisted of 32 items, including 22 single choice questions and 10 multiple choice questions, involving the following four parts: (a) dietary guidelines recommendations (items 1–18); (b) food and nutrients (items 19–22); (c) nutrition and disease prevention (items 23–26); and (d) nutrition skills (items 27–32). For single choice questions, a score of 1.5 (items 1–8, 19, 23, 27, 28) or 2.0 (items 9–13, 24, 29–32) was assigned to the correct answer, and 0 was assigned to the wrong or “don't know” answers. For multiple choice questions with 5 options (one option is the “don't know” answer, and at least two options are the correct answer), a score of 0 was assigned to the “don't know” answer, and 1.5 was assigned to each correct answer. Besides, if the wrong answer was not selected, 1.5 points will be further added, with a total score of 6.0 (except for item 18). The specific scoring criteria was shown in Supplementary Table S2. Further, the scores of all items were added up to calculate individual awareness levels of nutrition and health knowledge, ranging from 0 to 100. A total score of 75.0 and above was defined as “awareness”, otherwise defined as “unawareness”.

For multiple choice questions, we defined both choosing the correct answer and not choosing the wrong answer as knowing this option. To assess the responders' awareness of each multiple choice questions, we also calculated the proportion of knowing 0, 1, 2, 3 and 4 options.

Definition of analysis indicators as follows:

Awareness rate of each item (single choice question):


$$\begin{array}{l} \frac{the\;number\;of\;responders\;who\;selected\;the\;correct\;answer}{total\;number\;of\;responders\;who\;completed\;the\;survey}\times 100\% \end{array}$$

Awareness rate of each item (multiple choice questions):


$$\begin{array}{l} \frac{\begin{array}{c} the\;number\;of\;responders\;who\;were\;aware\;of\;3\;or\;more\\ options\;in\;each\;item \end{array}}{total\;number\;of\;responders\;who\;completed\;the\;survey}\times 100\% \end{array}$$

Awareness rate in each part:


$$\begin{array}{l} \frac{\begin{array}{c} \sum the\;number\;of\;responders\;who\;were\;aware\\ of\;each\;item \end{array}}{\begin{array}{c} total\;number\;of\;responders\;who\;completed\;the\;survey\\ \times total\;number\;of\;items\;in\;each\;part \end{array}}\times 100\% \end{array}$$

The total awareness rate:


$$\begin{array}{l} \frac{the\;number\;of\;responders\;with\;a\;total\;score\;of\;75\;or\;above}{total\;number\;of\;responders\;who\;completed\;the\;survey}\times 100\% \end{array}$$

### Covariates

Age was divided into five groups (≤24, 25–34, 35–44, 45–54, and 55–64 years). Gender was divided into two categories (male and female). Education level was allocated into six categories (primary school diploma or below, junior school diploma, high school diploma, junior college diploma, bachelor's degree, and master's degree or above). The occupation was allocated into five categories (medical workers, catering service workers, other health-related workers, educational workers, and others). The residential address included the downtown area and the remote area. A history of chronic diseases (including hypertension, diabetes, stroke, coronary heart disease, dyslipidemia, or other chronic diseases) was divided into three categories (Yes/No/Don't know).

### Quality control

The questionnaire was revised after review by an expert group, preliminary investigation and reliability and validity test. Prior to the online survey, the investigators at each monitoring site must receive professional training and assessment. During the survey, all investigators carried out the investigation strictly in accordance with the unified survey manuals, and the participants should be informed of the purpose and filling method prior to answering the questionnaire. After completing a questionnaire, an on-site inspection is conducted immediately. Additionally, after the investigation of all monitoring sites is completed, the quality control personnel of the Centers for Disease Control and Prevention will conduct a telephone return visit to the respondents according to the specially designed review questionnaire. If there are 3 or more unqualified questionnaires at one monitoring site, the on-site investigation was considered unqualified and should be re-investigated.

### Statistical analysis

The sociodemographic characteristics of respondents were all set as categorical variables and expressed as frequencies (percentages). Chi-square tests were used to analyze the associations between the total awareness rate of nutrition and health knowledge and sociodemographic characteristics. The awareness level of nutrition and health knowledge was a continuous variable with skewed distribution, presented as median (lower and upper quartile) and the differences between the two groups were compared using the Mann-Whitney U test. Multiple linear regression models were used to analyze the influencing factors of nutrition and health awareness. All statistical analyses were performed using SPSS version 25.0 (IBM Corporation). A two-tailed value of *p* < 0.05 was considered indicative of statistical significance.

## Results

### Sociodemographic characteristics of respondents

The average age of 33,436 respondents was (37.76 ± 12.29) years. 9430 (28.2%) were aged 35–44 years, and 7393 (22.1%) were aged 25–34 years. Males accounted for 47.0% and females for 53.0%. In terms of education level, 6.5% of respondents had a master's degree and 28.1% had a bachelor's degree or above. For the remainder, 23.7, 23.5, 15.6, and 2.7% of respondents had junior college, high school, junior school, and primary school diplomas or below respectively. In terms of occupation, respondents working in medical institutions, catering industry, other health-related industries, educational institutions and other work units accounted for 11.2, 4.0, 1.9, 8.5, and 74.4%, respectively. More than half of the responders (51.5%) were resident in the downtown city while the rest (48.5%) were resident in the far city. Additionally, respondents with chronic history accounted for 20.5% (Table 1).

**Table 1:** Sociodemographic characteristics of responders.

**Sociodemographic characteristics**	**Frequency (*n*)**	**Percentage (%)**
**Age (years)**		
18–24	6,444	19.3
25–34	7,393	22.1
35–44	9,430	28.2
45–54	6,521	19.5
55–64	3,648	10.9
**Gender**		
Male	15,724	47.0
Female	17,712	53.0
**Education level**		
Primary school diploma or below	893	2.7
Junior school diploma	5,206	15.6
High school diploma	7,845	23.5
Junior college diploma	7,916	23.7
Bachelor's degree	9,391	28.1
Master's degree or above	2,185	6.5
**Occupation**		
Medical workers	3,760	11.2
Catering service workers	1,342	4.0
Other health–related workers	624	1.9
Educational workers	2,848	8.5
Others	24,862	74.4
**Residential address**		
Downtown area	16,201	48.5
Remote area	17,235	51.5
**History of chronic disease**		
No	14,715	44.0
Yes	6,848	20.5
Don't know	11,873	35.5

### Awareness rate of nutrition and health knowledge among respondents

Among the four parts of nutrition and health knowledge, the part of nutrition and disease prevention knowledge had the highest awareness rate of 72.7%, followed by the knowledge of core recommendations of dietary guidelines (59.6%), food and nutrients (49.4%), and nutrition skills (46.3%). The top three items for the awareness rate in single choice questions were: Q12 (84.3%), Q23 (82.6%), and Q27 (80.6%). The bottom three single choice questions were Q29 (21.5%), Q10 (21.6%) and Q11 (28.7%). The top three items for the awareness rate in multiple choice questions were: Q18 (80.9%), Q17 (78.3%), and Q14 (71.0%). The bottom three items for the awareness rate in multiple choice questions were: Q21 (21.0%), Q22 (35.2%), and Q15 (56.8%) (Table 2). Besides, for nutrition and health knowledge multiple choice questions, we calculated the proportion of knowing 0, 1, 2, 3, and 4 options, respectively. The percentage of responders who got all the answers correct ranged from 2.4 to 57.9% (Supplementary Table S3).

**Table 2:** Awareness rate of nutrition and health knowledge of respondents.

**The items of nutrition and health knowledge**	**Awareness rate (*n*, %)**
**Core recommendations of dietary guidelines**	358,806 (59.6)
Q1. Recommendations on vegetable intake in the Dietary Guidelines for Chinese Residents (2016)	25,897 (77.5)
Q2. Recommendations on fruit intake in the Dietary Guidelines for Chinese Residents (2016)	20,897 (62.5)
Q3. Recommendations on dairy products intake in the Dietary Guidelines for Chinese Residents (2016)	13,335 (39.9)
Q4. Recommendations on soybean and its products intake in the Dietary Guidelines for Chinese Residents (2016)	9,719 (29.1)
Q5. Recommendations on meat intake in the Dietary Guidelines for Chinese Residents (2016)	22,981 (68.7)
Q6. Recommendations on egg intake in the Dietary Guidelines for Chinese Residents (2016)	24,001 (71.8)
Q7. Recommendations on processed meat intake in the Dietary Guidelines for Chinese Residents (2016)	22,462 (67.2)
Q8. Recommendations on sweet foods or beverage intake in the Dietary Guidelines for Chinese Residents (2016)	25,303 (75.7)
Q9. How much salt is recommended for healthy adults every day?	19,644 (58.8)
Q10. How much added sugar is recommended for healthy adults every day?	7,227 (21.6)
Q11. How much cooking oil is recommended for healthy adults every day?	9,591 (28.7)
Q12. Which of the following is more nutritious for lunch?	28,173 (84.3)
Q13. If an adult's body mass index is 26.1 kg/m^2^, what is his/her weight classification?	11,312 (33.8)
Q14. Which of the following statements about vegetables and fruits are true? *	23,754 (71.0)
Q15. Which of the following statements about Dietary Guidelines for Chinese Residents are true? *	18,986 (56.8)
Q16. Which of the following can help maintain a healthy weight? *	22,273 (66.6)
Q17. Which of the following are the correct explanations for saving food? *	26,204 (78.3)
Q18. Which of the following are the correct explanations for dietary hygiene? *	27,047 (80.9)
**Food and nutrients**	66,088 (49.4)
Q19. Which food is best for supplementing calcium?	24,570 (73.5)
Q20. Compared with refined staple foods, what are the nutritional values of coarse cereals? *	22,723 (68.0)
Q21. Which of the following foods is rich in iron and is easily absorbed by the body? *	7,020 (21.0)
Q22. Which foods below can supplement vitamin A? *	11,775 (35.2)
**Nutrition and disease prevention**	97,200 (72.7)
Q23. Which food contains more cooking oil and salt?	27,623 (82.6)
Q24. Which food is most beneficial to prevent dyslipidemia and cardiovascular disease?	26,108 (78.1)
Q25. Which of the following statements about salt/sugared beverages and chronic disease are true? *	19,735 (59.0)
Q26. Which of the following statements about foods and chronic disease are true? *	23,734 (71.0)
**Nutrition skills**	92,969 (46.3)
Q27. Read the food labels below. Which product contains more protein?	26,939 (80.6)
Q28. Read the food labels below. Which product belong to dairy products?	21,291 (63.7)
Q29. How much does a handful of vegetables weigh?	7,192 (21.5)
Q30. How much does a palm-sized piece of lean meat weigh?	10,061 (30.1)
Q31. How much does an ordinary egg weigh?	16,632 (49.7)
Q32. How much does a fist-sized steamed bread weigh?	10,854 (32.5)

^*^Multiple choice questions with 5 options (one option is the “don't know” answer, and at least two options are the correct answer). We defined both choosing the correct answer and not choosing the wrong answer as knowing this option. Responders are defined as knowing an item if they are aware of 3 or more options for this item.

### Analysis of the influencing factors of nutrition and health knowledge

There were 6,825 responders whose total score of nutrition and health knowledge was 75 or above, with a total awareness rate of 20.4% (6,825/33,436). Across the age groups, the awareness rate of responders aged 35–44 years was the highest (23.3%), while responders aged 18–24 years had the lowest awareness rate (17.6%). A significant association was found between age and the nutrition awareness rate (χ^2^ = 90.519, *p* < 0.001). A higher proportion of the female respondents (22.8%) were aware of nutrition and health knowledge compared with the male respondents (17.7%) (χ^2^ = 129.495, *p* < 0.001). The results also showed that there was a significant correlation between the awareness rate of nutrition and health knowledge and education level (χ^2^ = 715.809, *p* < 0.001), occupation (χ^2^ = 115.568, *p* < 0.001), residential address (χ^2^ = 140.783, *p* < 0.001), and a history of chronic disease (χ^2^ = 338.625, *p* < 0.001) (Table 3).

**Table 3:** Correlation of respondents' sociodemographic characteristics and the awareness rate of nutrition and health knowledge.

**Basic characteristics**	**Awareness rate (*n*, %)**	**χ^2^**	***p-*value**
**Age (years)**		90.519	<0.001
18–24	1,133 (17.6)		
25–34	1,511 (20.4)		
35–44	2,193 (23.3)		
45–54	1,327 (20.3)		
55–64	661 (18.1)		
**Gender**		129.495	<0.001
Male	2,791 (17.7)		
Female	4,034 (22.8)		
**Education level**		715.809	<0.001
Primary school diploma or below	96 (10.8)		
Junior school diploma	703 (13.5)		
High school diploma	1,292 (16.5)		
Junior college diploma	1,548 (19.6)		
Bachelor's degree	2,440 (26.0)		
Master's degree or above	746 (34.1)		
**Occupation**		115.568	<0.001
**Medical workers**	835 (22.2)		
Catering service workers	169 (12.6)		
Other health–related workers	79 (12.7)		
Educational workers	707 (24.8)		
**Others**	5,035 (20.3)		
**Residential address**		140.783	<0.001
Downtown area	3,744 (23.1)		
Remote area	3,081 (17.9)		
**History of chronic disease**		338.625	<0.001
No	3,618 (24.6)		
Yes	1,373 (20.0)		
Don't know	1,834 (15.4)		

Socio-demographic factors influencing the awareness level of nutrition and health knowledge were presented in Table 4. The final multivariable model showed that compared with responders aged 18–24 years, the awareness level was higher in the older group (β = 3.939, *p* < 0.001). Females were more aware of nutrition and health knowledge than males (β = 2.780, *p* < 0.001). Responders with higher education levels were more likely to have higher awareness levels (β = 15.259, *p* < 0.001). Compared with medical workers, catering service workers (β = −1.355, *p* = 0.001) and other health-related workers (β = −4.727, *p* < 0.001) had lower awareness levels, while educational workers (β = 2.059, *p* < 0.001) and other workers (β = 2.303, *p* < 0.001) had higher awareness levels. The awareness level of residents in a remote area was lower than that of downtown residents (β = −0.691, *p* < 0.001). Besides, compared with those without a history of chronic disease, those with chronic disease and those who did not know whether they had a chronic disease had a lower awareness level (Table 4).

**Table 4:** Factors influencing the awareness level of nutrition and health knowledge^*^.

**Variables**	**Awareness level [M (P25, P75)]**	**Univariable model**		**Multivariable model**	
		**β (95% CI)**	***p–*value**	**β (95% CI)**	***p-*value**
**Age (years)**					
18–24	64.5 (53.5, 72.0)	ref.		ref.	
25–34	66.0 (55.0, 73.5)	1.262 (0.799, 1.724)	<0.001	1.432 (0.989, 1.875)	<0.001
35–44	66.5 (55.0, 74.0)	1.780 (1.342, 2.219)	<0.001	2.937 (2.509, 3.365)	<0.001
45–54	65.5 (55.0, 73.5)	1.337 (0.861, 1.814)	<0.001	4.339 (3.848, 4.831)	<0.001
55–64	64.0 (53.0, 72.5)	−0.215 (−0.778, 0.347)	0.453	3.939 (3.344, 4.535)	<0.001
**Gender**					
Male	64.0 (52.5, 72.0)	ref.		ref.	
Female	67.0 (56.5, 74.0)	2.871 (2.575, 3.167)	<0.001	2.780 (2.496, 3.064)	<0.001
**Education level**					
Primary school diploma or below	54.5 (42.0, 67.5)	ref.		ref.	
Junior school diploma	61.5 (49.5, 70.0)	0.028 (−0.001, 0.056)	0.057	5.336 (4.398, 6.273)	<0.001
High school diploma	64.0 (53.0, 71.5)	0.057 (0.030, 0.085)	<0.001	8.202 (7.280, 9.123)	<0.001
Junior college diploma	65.0 (55.0, 73.0)	0.088 (0.060, 0.116)	<0.001	10.012 (9.075, 10.948)	<0.001
Bachelor's degree	68.0 (59.0, 75.0)	0.152 (0.125, 0.180)	<0.001	13.024 (12.083, 13.965)	<0.001
Master's degree or above	71.5 (63.5, 77.0)	0.234 (0.203, 0.265)	<0.001	15.259 (14.188, 16.330)	<0.001
**Occupation**					
Medical workers	65.0 (50.5, 74.0)	ref.		ref.	
Catering service workers	61.0 (47.5, 69.0)	−4.251 (−5.109, −3.392)	<0.001	−1.355 (−2.186, −0.524)	0.001
Other health–related workers	58.5 (43.6, 70.5)	−5.854 (−7.023, −4.685)	<0.001	−4.727 (−5.842, −3.611)	<0.001
Educational workers	68.0 (59.5, 74.5)	3.424 (2.763, 4.086)	<0.001	2.059 (1.411, 2.707)	<0.001
Others	65.5 (55.0, 73.0)	1.029 (0.571, 1.487)	<0.001	2.303 (1.860, 2.745)	<0.001
**Residential address**					
Downtown area	67.0 (56.5, 74.0)	ref.		ref.	
Remote area	64.0 (53.0, 72.0)	−2.256 (−2.552, −1.959)	<0.001	−0.691 (−0.984, −0.399)	<0.001
**History of chronic disease**					
No	67.5 (58.5, 74.5)	ref.		ref.	
Yes	64.5 (52.0, 73.0)	−3.745 (−4.137, −3.354)	<0.001	−3.666 (−4.062, −3.270)	<0.001
Don't know	63.0 (50.5, 71.5)	−5.287 (−5.617, −4.956)	<0.001	−5.143 (−5.462, −4.824)	<0.001

^*^Values are linear regression coefficients and 95% CIs. Only variables with statistically significant in univariate model were included in multivariate analysis.

## Discussion

Based on the China Nutrition and Health Knowledge Survey, we conducted a large-scale cross-sectional study to comprehensively assess the nutrition and health knowledge level of Wuhan residents, and to examine how awareness levels varied across sociodemographic characteristics. Our results showed that the total awareness rate of nutrition and health knowledge of Wuhan residents was not optimistic (20.4%), especially in nutrition skills and food and nutrients aspects. Besides, we found individual sociodemographic characteristics, including age, gender, education level, occupation, residential address, and the history of chronic disease, were significantly associated with their awareness level of nutrition and health knowledge, suggesting that divergent demographic characteristics should be considered in carrying out various forms of nutrition education and promotional activities.

The awareness rate of nutrition and health knowledge in this study was similar to the results of the survey among adults in 9 provinces in China (21.1%), but much lower than that of adult residents in 3 municipalities (Beijing, Shanghai and Chongqing; 45.0%) (26), Suzhou (52.3%) (27) and Zhengzhou (40.5%) (28). Compared with foreign countries, the awareness rate of Wuhan residents is also lower than that of adults in the three central cities and rural areas of Italy (46.0%) (29) and the adults aged≥ 35 years in Central and Southern Italy (30). These discrepancies could be partially attributed to differences in study participants (race, economic status, age composition, etc.), questionnaire composition, and definition of nutrition awareness. For one-third of the items, nevertheless, about half of the respondents could not answer correctly. These misconceptions may further lead to inaccurate nutritional skills and undesirable dietary behaviors (7, 31). As Dickson-Spillmann et al. (32) indicated, for instance, participants with a lower level of nutrition knowledge consumed fewer fruits and vegetables, but more sausages than those with higher nutrition knowledge. Among the four parts of nutrition and health knowledge, we found respondents had the lowest overall awareness in nutrition skills (46.3%), especially when it came to estimating food weight (items Q29–Q32), with awareness rates ranging from 21.5 to 49.7%. These results suggested that respondents do not have a good grasp of the skills of estimating food portions, which may naturally lead to overconsumption or underconsumption, resulting in a massive gap between recommended intake and actual intake. Due to the complex interaction between nutritional information processing, motivational factors and behavior change, there is a significant gap between perceived knowledge and practical dietary intake (33, 34). Despite this, it is widely acknowledged that popularizing nutrition knowledge, including but not limited to nutritional skills, is essential.

In terms of food and nutrition, the awareness rate of each item ranged from 21.0 to 73.5%. The lower awareness rate of items 21 and 22 highlights the fact that the nutritional value of food is not present in many responders' minds and is not factored into their daily food choices. Therefore, more efforts are needed to increase people's awareness of food nutrition in order to facilitate people to choose food more rationally.

Given that awareness of dietary guidelines is a key factor in whether people are likely to adopt the recommended behavior, adherence to dietary guidelines may be particularly valuable in promoting rational dietary intake and reducing the risk of nutrition-related chronic disease (5, 35, 36), more than half of the items in this study are related to the core recommendations of Dietary Guidelines for Chinese Residents. The overall awareness rate of core recommendations of dietary guidelines in this study was 59.6%. However, the awareness rate of the recommended intake of added sugar (Q10), cooking oil (Q11), and the classification of body mass index (Q13) was lower than or close to 30.0%, indicating that responders were insensitive to number-based dietary recommendations, which may result in excessive intake of added sugar and cooking oil, and even increase the risk of diabetes and obesity (37, 38). In addition, we found that, compared with the awareness about the recommended intake of vegetables (Q1), eggs (Q6), meats (Q5 and Q7), and fruits (Q2), responders had a lower awareness about the recommended intake of soybean and its products (Q4) and dairy products (Q3), which tend to be the most overlooked daily foods. In this regard, it is suggested to emphasize the recommended intake of soybean and its products and dairy products, as well as the adverse effects caused by their deficiencies when carrying out the education about Dietary Guidelines for Chinese Residents. In respect of nutrition and disease prevention, the responders demonstrated higher awareness (72.7%), suggesting that although the respondents had a clear comprehension of diet related to possible chronic diseases, they could not regulate their daily diet well. Consistently, Bullen et al. (39) revealed that those who acquired factual knowledge did not make good use of their knowledge to change their unhealthy eating behavior. Generally speaking, although nutrition awareness is not always associated with a distinct dietary behavior, we still need to persist in popularizing people's nutrition and health knowledge from multiple perspectives. Specifically, in the future, it is suggested to disseminate rich and easy to accept nutrition and health knowledge to the population from multiple perspectives such as promoting dietary guidelines and nutrition skills training through various forms, for example, dietary training, expert consultation and thematic discussion.

A considerable amount of literature has been published on the relationship between sociodemographic characteristics and nutrition knowledge (40, 41). Similar to the findings in other studies (23, 42), we found females tended to have higher nutrition awareness than males, which might be related to the fact that Chinese women mostly assume the roles of housewives and thus master more nutrition-related knowledge than men. Previous studies on the relationship between age and nutrition awareness have been equivocal (22, 23, 42). Compared to responders aged 18–24 years, we found the older tended to be more nutritionally aware, among which the middle-aged and elderly showed the highest nutrition awareness. Similarly, Glanz et al. (42) showed that the elderly were more nutritionally conscious than younger people. Plausible explanations for these results might be that older people have greater access to nutrition education activities. Conversely, the National Health and Nutrition Examination Survey 2005–2006 reported that the younger were more likely to have a higher nutrition awareness rate than the older (22), while Girois et al. (23) did not report any associations between age and nutrition awareness. However, divergent measures of nutrition awareness used in these comparisons should be taken into consideration. Several previous studies pointed out that individual nutrition level was positively correlated with their education level (17, 41), which was consistent with our findings. Moreover, we found medical workers and educational workers tended to be more aware of nutrition knowledge than other professions. The possible explanation for this result might be that the higher the level of education, the greater the learning capacity and the more opportunities for nutrition information. Besides, our study indicated that responders without the chronic disease had higher awareness levels, which may be related to their more attention to health care and nutrition information. In turn, those who do not know whether they have chronic diseases may also be indifferent to nutrition and health knowledge (43). Last but not least, our findings highlight the need to strengthen nutrition education for responders living in the remote area, as their access to nutrition information and basic public health services is significantly lower than that of responders living in the downtown area. The above findings of this study suggested that nutrition and health knowledge level varies across populations with various demographic characteristics. In the future nutrition campaign, we should give full play to the role of professional teams such as medical personnel, nutritionists and educators, and go deep into communities, rural areas, schools and other places to provide targeted dietary guidance for those younger, less educated, living in remote areas and with weak nutrition awareness.

Our study has several strengths. Firstly, the large sample size and representative study population came from 15 monitoring sites in Wuhan, which provided strong evidence for examining the nutrition awareness level of Wuhan residents. Secondly, we used validated questionnaires to evaluate participants' awareness of nutrition and health knowledge from various aspects. The results of our study can provide a reference for future nutrition questionnaire design. Finally, we have adopted strict quality control before, during and after the investigation to minimize potential deviations.

Our study also has some potential limitations. Firstly, since this survey uses a self-administered questionnaire, there might be some cases where responders filled in the questionnaire carelessly, however, three-level quality control measures were taken to reduce the information bias as much as possible. Secondly, although the participants in this study were representative of the Wuhan population in terms of age, gender, and residential area, we could not rule out the potential sampling bias. Thirdly, various definitions of nutrition awareness may contribute to different results, therefore, the results of interpolation and extrapolation need to be treated with caution. Last but not least, different scoring criteria and questionnaire design, such as the representative questions selected in each part may contribute to different results. However, in order to acquire the shortcoming of residents' nutrition and health knowledge in a large scale, the questions designed in each part should not only be closely related to nutrition and health, but also easy to understandable and representative. Additionally, the questionnaire used in this study were determined by expert review, pre-survey, reliability and validity test, which can ensure the design quality of the questionnaire.

## Conclusion

In conclusion, in this large-scale cross-sectional study, the total awareness rate of nutrition and health knowledge among Wuhan residents was far from reaching the national requirements, especially in terms of nutrition skills. Besides, we found evidence that sociodemographic characteristics were associated with nutrition and health knowledge. Further nutrition education campaign is needed to target certain subgroups of the population or specific nutrition concerns.

## Data availability statement

The original contributions presented in the study are included in the article/Supplementary material, further inquiries can be directed to the corresponding author/s.

## Author contributions

YW, SW, MS, LH, and XW designed the research. LH and XW supervised the study conduct. YW, SW, MS, XW, HL, SG, and LT conducted the research. SW analyzed the data and wrote the manuscript. YW and MS coedited, revised, and finally reviewed the manuscript critically for important intellectual content. All the authors read and approved the final version of the manuscript.

## Funding

This work was supported by the DSM-Chinese Nutrition Society Science and Technology Award (CNS-DSM2018A34).

## Conflict of interest

The authors declare that the research was conducted in the absence of any commercial or financial relationships that could be construed as a potential conflict of interest.

## Publisher's note

All claims expressed in this article are solely those of the authors and do not necessarily represent those of their affiliated organizations, or those of the publisher, the editors and the reviewers. Any product that may be evaluated in this article, or claim that may be made by its manufacturer, is not guaranteed or endorsed by the publisher.
