# Supplementary material for: Awareness of nutrition and health knowledge and its influencing factors among Wuhan residents
Source: Front Public Health. 2022 Oct 5;10:987755. doi: 10.3389/fpubh.2022.987755 (PMC9580461; doi:10.3389/fpubh.2022.987755)
Supplement: Supplementary file 1 [file Table_1.docx]

***Supplement Table S1*** Actual survey distribution at each monitoring site

| Monitoring site | Community (n) | Responders (n) | Constituent ratio (%) |
| --- | --- | --- | --- |
| Jianghan District | 6 | 1829 | 5.5 |
| Qiaokou District | 6 | 1946 | 5.8 |
| Hanyang District | 7 | 2528 | 7.6 |
| Wuchang District | 10 | 3304 | 9.9 |
| Hongshan District | 15 | 4586 | 13.7 |
| Qingshan District | 4 | 1368 | 4.1 |
| Jiang’an District | 1 | 330 | 1.0 |
| East Lake Scenic Area | 1 | 310 | 0.9 |
| Hannan District | 4 | 1422 | 4.3 |
| Dongxihu District | 8 | 2638 | 7.9 |
| Caidian District | 5 | 1646 | 4.9 |
| Huangpi District | 10 | 3238 | 9.7 |
| Jiangxia District | 9 | 2839 | 8.5 |
| Xinzhou District | 8 | 2825 | 8.4 |
| East Lake High-tech Development Zone | 8 | 2627 | 7.9 |
