# Supplementary material for: Awareness of nutrition and health knowledge and its influencing factors among Wuhan residents
Source: Front Public Health. 2022 Oct 5;10:987755. doi: 10.3389/fpubh.2022.987755 (PMC9580461; doi:10.3389/fpubh.2022.987755)
Supplement: Supplementary file 2 [file Table_2.docx]

***Supplementary Table S2*** Scoring criteria for the items of nutrition and health knowledge

| Items of nutrition and health knowledge | Score ranges | Scoring criteria |
| --- | --- | --- |
| Core recommendations of dietary guidelines |  |  |
| Q1. Recommendations on vegetable intake in the Dietary Guidelines for Chinese Residents (2016) | 0-1.5 | 1.5 points for a correct answer and 0 for a wrong answer |
| Q2. Recommendations on fruit intake in the Dietary Guidelines for Chinese Residents (2016) | 0-1.5 |  |
| Q3. Recommendations on dairy products intake in the Dietary Guidelines for Chinese Residents (2016) | 0-1.5 |  |
| Q4. Recommendations on soybean and its products intake in the Dietary Guidelines for Chinese Residents (2016) | 0-1.5 |  |
| Q5. Recommendations on meat intake in the Dietary Guidelines for Chinese Residents (2016) | 0-1.5 |  |
| Q6. Recommendations on egg intake in the Dietary Guidelines for Chinese Residents (2016) | 0-1.5 |  |
| Q7. Recommendations on processed meat intake in the Dietary Guidelines for Chinese Residents (2016) | 0-1.5 |  |
| Q8. Recommendations on sweet foods or beverage intake in the Dietary Guidelines for Chinese Residents (2016) | 0-1.5 |  |
| Q9. How much salt is recommended for healthy adults every day? | 0-2 | 2 points for a correct answer and 0 for a wrong answer |
| Q10. How much added sugar is recommended for healthy adults every day? | 0-2 |  |
| Q11. How much cooking oil is recommended for healthy adults every day? | 0-2 |  |
| Q12. Which of the following is more nutritious for lunch? | 0-2 |  |
| Q13. If an adult’s body mass index is 26.1 kg/m^2^, what is his/her weight classification? | 0-2 |  |
| Q14. Which of the following statements about vegetables and fruits are true? ^*^ | 0-6 | There are totally 5 options. A score of 0 was assigned to “don’t know” answer, 1.5 was assigned to each correct answer. Besides, if a wrong option was not selected, 1.5 points will be added, with a total score of 6. |
| Q15. Which of the following statements about Dietary Guidelines for Chinese Residents are true? ^*^ | 0-6 |  |
| Q16. Which of the following can help maintain a healthy weight? ^*^ | 0-6 |  |
| Q17. Which of the following are the correct explanations for saving food? ^*^ | 0-6 |  |
| Q18. Which of the following are the correct explanations for dietary hygiene? ^*^ | 0-8 | There are totally 5 options. A score of 0 was assigned to “don’t know” answer, 2 was assigned to each correct answer. Besides, if a wrong option was not selected, 2 points will be added, with a total score of 8. |
| Food and nutrients |  |  |
| Q19. Which food is best for supplementing calcium? | 0-1.5 | 1.5 points for a correct answer and 0 for a wrong answer |
| Q20. Compared with refined staple foods, what are the nutritional values of coarse cereals? ^*^ | 0-6 | There are totally 5 options. A score of 0 was assigned to “don’t know” answer, 1.5 was assigned to each correct answer. Besides, if a wrong option was not selected, 1.5 points will be added, with a total score of 6. |
| Q21. Which of the following foods is rich in iron and is easily absorbed by the body? ^*^ | 0-6 |  |
| Q22. Which foods below can supplement vitamin A? ^*^ | 0-6 |  |
| Nutrition and disease prevention |  |  |
| Q23. Which food contains more cooking oil and salt? | 0-1.5 | 1.5 points for a correct answer and 0 for a wrong answer |
| Q24. Which food is most beneficial to prevent dyslipidemia and cardiovascular disease? | 0-2 | 2.0 points for a correct answer and 0 for a wrong answer |
| Q25. Which of the following statements about salt/sugared beverages and chronic disease are true? ^*^ | 0-6 | There are totally 5 options. A score of 0 was assigned to “don’t know” answer, 1.5 was assigned to each correct answer. Besides, if a wrong option was not selected, 1.5 points will be added, with a total score of 6. |
| Q26. Which of the following statements about foods and chronic disease are true? ^*^ | 0-6 |  |
| Nutrition skills |  |  |
| Q27. Read the food labels below. Which product contains more protein? | 0-1.5 | 1.5 points for a correct answer and 0 for a wrong answer |
| Q28. Read the food labels below. Which product belong to dairy products? | 0-1.5 |  |
| Q29. How much does a handful of vegetables weigh？ | 0-2 | 2 points for a correct answer and 0 for a wrong answer |
| Q30. How much does a palm-sized piece of lean meat weigh？ | 0-2 |  |
| Q31. How much does an ordinary egg weigh? | 0-2 |  |
| Q32. How much does a fist-sized steamed bread weigh? | 0-2 |  |
| Total scores | 0-100 |  |
