# Supplementary material for: Awareness of nutrition and health knowledge and its influencing factors among Wuhan residents
Source: Front Public Health. 2022 Oct 5;10:987755. doi: 10.3389/fpubh.2022.987755 (PMC9580461; doi:10.3389/fpubh.2022.987755)
Supplement: Supplementary file 3 [file Table_3.docx]

***Supplementary Table S3*** Awareness rate of nutrition and health knowledge in multiple choice question ^*^

| Items of nutrition and health knowledge | Number of options known | | | | |
| --- | --- | --- | --- | --- | --- |
|  | 0 | 1 | 2 | 3 | 4 |
| Core recommendations of dietary guidelines |  |  |  |  |  |
| Q14. Which of the following statements about vegetables and fruits are true? | 490 (1.5) | 2582 (7.7) | 6610 (19.8) | 12771 (38.2) | 10983 (32.8) |
| Q15. Which of the following statements about Dietary Guidelines for Chinese Residents are true? | 886 (2.6) | 4701 (14.1) | 8863 (26.5) | 10621 (31.8) | 8365 (25.0) |
| Q16. Which of the following can help maintain a healthy weight? | 1132 (3.4) | 4452 (13.3) | 5579 (16.7) | 4277 (12.8) | 17996 (53.8) |
| Q17. Which of the following are the correct explanations for saving food? | 418 (1.3) | 1771 (5.3) | 5043 (15.1) | 10942 (32.7) | 15262 (45.6) |
| Q18. Which of the following are the correct explanations for dietary hygiene? | 238 (0.7) | 1238 (3.7) | 4913 (14.7) | 7687 (23.0) | 19360 (57.9) |
| Food and nutrients |  |  |  |  |  |
| Q20. Compared with refined staple foods, what are the nutritional values of coarse cereals? | 410 (1.2) | 2743 (8.2) | 7560 (22.6) | 13555 (40.5) | 9168 (27.4) |
| Q21. Which of the following foods is rich in iron and is easily absorbed by the body? | 3925 (11.7) | 12316 (36.8) | 10175 (30.4) | 6224 (18.6) | 796 (2.4) |
| Q22. Which foods below can supplement vitamin A? | 1866 (5.6) | 8139 (24.3) | 11656 (34.9) | 10831 (32.4) | 944 (2.8) |
| Nutrition and disease prevention |  |  |  |  |  |
| Q25. Which of the following statements about salt/sugared beverages and chronic disease are true? | 1195 (3.6) | 4000 (12.0) | 8506 (25.4) | 9047 (27.1) | 10688 (32.0) |
| Q26. Which of the following statements about foods and disease are true? | 370 (1.1) | 3046 (9.1) | 6286 (18.8) | 14756 (44.1) | 8978 (26.9) |

^*^ Multiple choice questions with 5 options (one option is the “don’t know” answer, and at least two options are the correct answer). We defined both choosing the correct answer and not choosing the wrong answer as knowing this option. Further, we calculated the proportion of knowing 0, 1, 2, 3 and 4 options.
